# Regional differences in annual prevalence of sustainable working life in Swedish twin cohort

**DOI:** 10.1186/s13104-023-06503-y

**Published:** 2023-09-21

**Authors:** Auriba Raza, Mo Wang, Jurgita Narusyte, Pia Svedberg, Annina Ropponen

**Affiliations:** 1 Division of Insurance Medicine, Department of Clinical Neuroscience, Karolinska Institutet, Stockholm, SE-171 77 Sweden; 2 Finnish Institute of Occupational Health, Työterveyslaitos, 00032 Finland

**Keywords:** Sustainable working life, Regional differences, Twins, Annual prevalence

## Abstract

The global rise in life expectancy transforms age structure consequently having impact to the sustainability of social protection systems and working life. This descriptive study aimed to illustrate the annual prevalence of sustainable working life across Swedish residential regions, and investigate differences between age groups, sex, or being identical or fraternal twin. The study sample included 81,231 twins with linkage to national register data on sickness absence, disability pension, unemployment, and residential regions. Regions were classified by Swedish municipalities into nine groups. Sustainable working life were then followed in 1998, 2003, 2008, and 2013. Annual prevalence and Generalized Estimating Equation (GEE) with log linear models for interaction test were used. Medium to large size municipalities (15–24%) had higher annual prevalence of sustainable working life than smaller municipalities (1–7%). Young adults in medium to large size municipalities had high annual prevalence of sustainable working life. We found no differences for sexes or being identical or fraternal twin. To conclude, annual prevalence of sustainable working life in 1998, 2003, 2008 and 2013 differed by region being highest in medium to large size municipalities which may have importance for targeting policies, regulations, and practices in a region-specific way. Further studies on residential regions and sustainable working life would be merited to confirm associations and other influential factors.

**Supplementary Information:**

The online version contains supplementary material available at 10.1186/s13104-023-06503-y.

## Introduction

The increase in sickness absence (SA) and disability pension (DP) and persistent unemployment remain an economical and public health concern in many Organization for Economic Cooperation and Development (OECD) countries [1–3] and calls for sustainable working life, i.e., longer working lives, and healthy life years [4]. Sustainable working life is a rather new concept defined as living and working conditions that support people in engaging and remaining in work throughout an extended working life [5, 6]. Living and working conditions can include wide range of aspects, e.g., health, physical/mental/psychosocial work environment, work motivation, family situation, leisure activities [7, 8] but also residential region [9–13]. Studies of regional differences for sustainable working life even within one country remain scarce [9–13]. A need exists to identify regional differences in sustainable working life to assist further studies but also consequently effective targeting of policies, regulations, and practices.

The theoretical framework of this study was based on the fact that living and working conditions are known to depend on compositional and contextual factors due to regional differences within a country [14]. E.g., this approach was due to known regional differences in SA/DP, or unemployment although being opposites of sustainable working life [9–13, 15–20]. Regional differences may be due to the differences in demographic composition of an area or alternatively due to socioeconomic circumstances [9–12], and out of demographic factors, higher age and being women have been associated with SA/DP [17, 18, 21, 22]. Thus, in the assessment of regional differences for sustainable working life, influential sociodemographic factors would merit attention especially in the Swedish context since sustainable working life is one of the government’s prioritized areas [23]. Furthermore, the global rise in life expectancy [24], especially in Nordic countries [25], has transformed and will continue to transform age structure in ways that might jeopardize the sustainability of social protection systems [5, 26]. Therefore, changes over time in social insurance regulations may affect regional differences in sustainable working life since they play an inevitable role for SA/DP and unemployment [27, 28]. Also, societal changes have taken place over time, e.g., the financial crisis in 2008 increased prevalence of unemployment across educational levels from 2009 [29]. Yet indications exist that national policies might have limited influence on modifying the associations between employment and health [30], but the societal effects may still remain [31]. Studies would be needed to clarify the regional differences since they would be needed to effectively target policies, regulations, and practices focused on promoting sustainable working life.

Besides the regions and sociodemographic factors, twin studies with possibility to assess the difference between identical vs. fraternal twins might add to the studies based on other populations. The comparison of identical vs. fraternal (same-sexed) twin pairs, provide preliminary estimates of familial effects (genetics and shared environment) on the factors of interest. In this descriptive study based on the Swedish Twin project of Disability pension and Sickness absence (STODS) [21] including twins identified through the Swedish Twin Registry [32] born between 1925 and 1990, we illustrated the annual prevalence of sustainable working life in different regions in Sweden. We further indicate differences in regional annual prevalence of sustainable working life by age groups, sex, and between identical and fraternal twins.

## Materials and methods

The study sample of STODS was restricted to twins who were alive, living in Sweden with data on regions, and 18–67 years old at baseline i.e., 31 December 1998 (n = 81,231 individuals). The baseline study sample included 19,109 identical (monozygotic) twins and 23,671 same-sexed fraternal (dizygotic) twins. These were followed in 2003 (n = 76,667), 2008 (n = 66,124), and 2013 (n = 54,871).

Sustainable working life was conceptualized as not having interruptions of working life due to unemployment, SA, or DP [21] and measured in years 1998, 2003, 2008, and 2013 since they included comprehensive data both for regions and sustainable working life. Furthermore, due to descriptive nature of this study, the five-year span between years was selected to limit the number of time points. The measure of sustainable working life was dichotomized (yes/no) for being employed in these years excluding any interruptions, based on the data of DP (date) and SA (date of when each SA spell began and ended) for respective years and were collected from the Swedish Social Insurance Agency MiDAS-database [33, 34]. Furthermore, data on unemployment, and emigration from Statistics Sweden (SCB) LISA database [35], and date of death was from the cause of death registry maintained by the National Board of Health and Welfare were used. Those who emigrated or died between the time points (i.e., 1998, 2003, 2008 and 2013) were censored.

Type of residential regions were derived from SCB’s LISA database [35] and we applied the classification of Swedish municipalities by the Swedish Association of Local Authorities and Regions (SALAR) to data [36]. The municipalities were categorized into nine groups based on structural parameters such as population and commuting patterns as has been done before [37–39]:

1: Large cities - municipalities with a population of at least 200,000 inhabitants with at least 200,000 inhabitants in the largest urban area.

2: Commuting municipalities near large cities – municipalities where more than 40% of the working population commute to work in a large city or municipality near a large city.

3: Medium-sized towns – municipalities with a population of at least 50 000 inhabitants with at least 40,000 inhabitants in the largest urban area.

4: Commuting municipalities near medium-sized towns - municipalities where more than 40% of the working population commute to work in a medium-sized town.

5: Commuting municipalities with a low commuting rate near medium-sized towns - municipalities where less than 40% of the working population commute to work in a medium-sized town.

6: Small towns - municipalities with a population of at least 15,000 inhabitants in the largest urban area.

7: Commuting municipalities near small towns - municipalities where more than 30% of the working population commute to work in a small town/ urban area or more than 30% of the employed day population lives in another municipality.

8: Rural municipalities - municipalities with a population of less than 15,000 inhabitants in the largest urban area, very low commuting rate (less than 30%).

9: Rural municipalities with a visitor industry – municipalities in rural area that fulfil at least two criteria for visitor industry, i.e., number of overnight stays, retail-, restaurant- or hotel turnover per head of population.

Analyses were stratified by, sex, age in five groups (18 to 27, 28 to 37, 38 to 47, 48 to 57, and 58 to 67 years); and zygosity (identical and fraternal same-sexed twins).

### Statistical analyses

A prospective cohort design for annual prevalence (reporting frequencies and percentages) of sustainable working life in different regions across Sweden in the four time points (1998, 2003, 2008, and 2013) was used. Annual prevalence of sustainable working life for each region was calculated by each follow-up year, stratified by sex, by age groups, and for potential effects of familial confounding (i.e., genetics and shared, childhood environment), by identical and same-sexed fraternal twins. Differences in annual prevalence across years in age groups within a region were tested using two-way interaction by Generalized Estimating Equation (GEE) with log linear models for repeated measures. All the analyses were performed with STATA, version 13.

## Results

At the baseline year 1998, our study sample consisted of 81,231 individuals, 50% women, and 25% were 48 to 57 years old (Table 1).

**Table 1:** Descriptive characteristics of the study population in each region and in total sample at the baseline year 1998

	Regions, n (%^*^)									Total N (%^¤^)
	1	2	3	4	5	6	7	8	9	
**Sex**										
Women	6339 (16)	6760 (16)	9815 (24)	3431 (8)	2940 (7)	5739 (14)	2734 (7)	2422 (6)	650 (2)	40 830 (50)
**Age categories**										
18–27	2652 (17)	2394 (15)	4163 (27)	1242 (8)	1021 (7)	2057 (13)	909 (6)	866 (6)	234 (2)	15 538 (19)
28–37	3203 (18)	2997 (17)	4088 (24)	1389 (8)	1094 (6)	2401 (14)	1049 (6)	913 (5)	245 (1)	17 379 (21)
38–47	2617 (15)	2848 (16)	3954 (23)	1602 (9)	1353 (8)	2566 (15)	1268 (7)	1095 (6)	283 (1)	17 586 (22)
48–57	2553 (13)	3384 (17)	4612 (23)	1817 (9)	1499 (7)	3007 (15)	1559 (8)	148 (7)	385 (2)	20 164 (25)
58–67	1231 (12)	1558 (15)	2395 (23)	988 (9)	819 (8)	1736 (16)	895 (5)	754 (7)	188 (29	10 564 (13)
**Zygosity** **€**										
Monozygotic	2878 (15)	3293 (17)	4665 (24)	1610 (8)	1302 (7)	2739 (14)	1260 (7)	1038 (5)	324 (2)	19 109 (24)
Dizygotic	3434 (15)	3781 (16)	5506 (23)	2107 (9)	1743 (7)	3493 (15)	1711 (7)	1508 (6)	388 (2)	23 671 (29)

^*^In region

^¤^In total study sample of 81 231 twins

**€**Monozygotic = identical twin, Dizygotic = same-sex fraternal twin, opposite-sexed dizygotic twins or twins without known zygosity not included

**Fig. 1:**
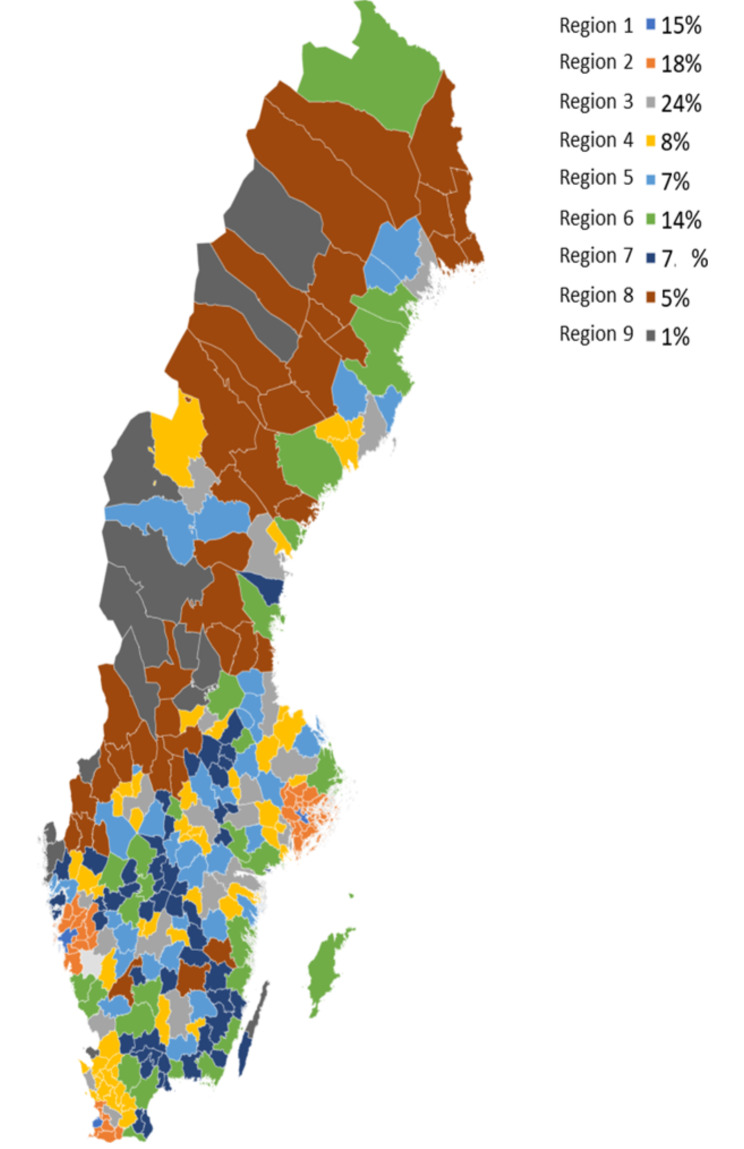
Annual prevalence of sustainable working life across different regions in Sweden in 1998; Region 1 Large cities - municipalities with a population of at least 200 000 inhabitants with at least 200 000 inhabitants in the largest urban area, Region 2 Commuting municipalities near large cities – municipalities where more than 40% of the working population commute to work in a large city or municipality near a large city, Region 3 Medium-sized towns – municipalities with a population of at least 50 000 inhabitants with at least 40 000 inhabitants in the largest urban area, Region 4 Commuting municipalities near medium-sized towns - municipalities where more than 40% of the working population commute to work in a medium-sized town, Region 5 Commuting municipalities with a low commuting rate near medium-sized towns - municipalities where less than 40% of the working population commute to work in a medium-sized town, Region 6 Small towns - municipalities with a population of at least 15 000 inhabitants in the largest urban area, Region 7 Commuting municipalities near small towns - municipalities where more than 30% of the working population commute to work in a small town/ urban area or more than 30% of the employed day population lives in another municipality, Region 8 Rural municipalities - municipalities with a population of less than 15 000 inhabitants in the largest urban area, very low commuting rate (less than 30%), and Region 9 Rural municipalities with a visitor industry – municipalities in rural area that fulfil at least two criteria for visitor industry, i.e. number of overnight stays, retail-, restaurant- or hotel turnover per head of population

Annual prevalence of sustainable working life was higher for medium to large size municipalities (15 to 24%) compared to smaller ones demonstrating low (1 to 7%) annual prevalence (Table 2; Fig. 1), the pattern being the same in the follow-up. Based on visual evaluation, women and men had similar annual prevalence of sustainable working life across regions (Supplemental Table 1), and among identical and same-sexed fraternal twins (data not shown).

**Table 2:** Follow up of individuals from the year 1998: Percentages^*^ of individuals with sustainable work life across different regions in Sweden. Information of individuals living in these regions and sustainable working life are from the years 1998, 2003, 2008, and 2013^#^

Classification of Swedish municipalities 2017 by Swedish Municipalities and Regions 9 (36)	1998 (n = 56,255)		2003 (n = 52,851)		2008 (n = 49,399)		2013 (n = 42,016)	
	n	%	n	%	n	%	n	%
1: Large cities - municipalities with a population of at least 200 000 inhabitants with at least 200 000 inhabitants in the largest urban area.	8680	15	8741	17	8259	17	6982	17
2: Commuting municipalities near large cities – municipalities where more than 40% of the working population commute to work in a large city or municipality near a large city.	9918	18	9340	18	8726	18	7775	19
3: Medium-sized towns – municipalities with a population of at least 50 000 inhabitants with at least 40 000 inhabitants in the largest urban area.	13,245	24	12,569	24	11,495	23	9583	23
4: Commuting municipalities near medium-sized towns - municipalities where more than 40% of the working population commute to work in a medium-sized town.	4709	8	4437	8	4227	9	3720	9
5: Commuting municipalities with a low commuting rate near medium-sized towns - municipalities where less than 40% of the working population commute to work in a medium-sized town.	3901	7	3585	7	3323	7	2797	7
6: Small towns - municipalities with a population of at least 15 000 inhabitants in the largest urban area.	8062	14	7274	14	6947	14	5854	14
7: Commuting municipalities near small towns - municipalities where more than 30% of the working population commute to work in a small town/ urban area or more than 30% of the employed day population lives in another municipality.	3841	7	3497	7	3124	6	2556	6
8: Rural municipalities - municipalities with a population of less than 15 000 inhabitants in the largest urban area, very low commuting rate (less than 30%)	3068	5	2637	5	2554	5	2105	5
9: Rural municipalities with a visitor industry – municipalities in rural area that fulfil at least two criteria for visitor industry, i.e., number of overnight stays, retail-, restaurant- or hotel turnover per head of population.	831	1	771	1	744	2	644	2

^*^Percentages might not sum up to 100 due to rounding

^#^The number of individuals in each year of follow-up is decreasing due to censoring

Among the age groups, annual prevalence of sustainable working life in the age group 18-27 years was higher in the follow-up years (2003, 2008) compared to the baseline (1998) in region one, however, it was lower in regions 2, 4, and 8 (*p*-value for interaction <0.03) (Table 3). Further, the annual prevalence in 2003 was significantly different from the previous year 1998 and the annual prevalence in 2008 was significantly different from the previous year 2003 in regions one and two in the age group 18-27 years (*p*-value for interaction <0.001 (Supplemental Fig. 1). Regions 3-5 and 8 showed similar patterns (Supplemental Fig. 1).

**Table 3:** **Follow up of individuals followed from the year 1998**: Percentage^*^ of individuals in different age groups with sustainable work life across different regions in Sweden. Information of individuals living in these regions and sustainable working life are from the year 1998, 2003, 2008, and 2013

Classification of Swedish municipalities 2017 by Swedish Municipalities and Regions 9 (36)	Year	Age categories (years of age)				
		18–27 (n = 20,187)^¤^	28–37 (n = 44,544)	38–47 (n = 51,761)	48–57 (n = 52,261)	58–67 (n = 31,768)
		%	%	%	%	%
1: Large cities - municipalities with a population of at least 200 000 inhabitants with at least 200 000 inhabitants in the largest urban area.	1998^#^	18	19	15	12	12
	2003^#^	21	20	15	14	13
	2008^#^	27	22	15	15	12
	2013	-	23	16	15	14
2: Commuting municipalities near large cities – municipalities where more than 40% of the working population commute to work in a large city or municipality near a large city.	1998^#^	17	19	17	18	16
	2003^#^	15	18	19	17	18
	2008^#^	14	18	20	17	16
	2013	-	18	21	19	15
3: Medium-sized towns – municipalities with a population of at least 50 000 inhabitants with at least 40 000 inhabitants in the largest urban area.	1998	27	23	23	23	22
	2003	31	24	22	22	22
	2008	26	24	23	23	23
	2013	-	24	23	23	22
4: Commuting municipalities near medium-sized towns - municipalities where more than 40% of the working population commute to work in a medium-sized town.	1998	8	6	9	9	9
	2003	6	8	9	9	9
	2008	6	8	9	9	10
	2013	-	8	9	9	9
5: Commuting municipalities with a low commuting rate near medium-sized towns - municipalities where less than 40% of the working population commute to work in a medium-sized town.	1998	6	6	8	7	8
	2003	5	6	7	8	7
	2008	5	6	7	8	8
	2013	-	5	6	7	8
6: Small towns - municipalities with a population of at least 15 000 inhabitants in the largest urban area.	1998	13	14	15	15	17
	2003	12	12	14	15	15
	2008	13	12	14	15	15
	2013	-	12	13	14	16
7: Commuting municipalities near small towns - municipalities where more than 30% of the working population commute to work in a small town/ urban area or more than 30% of the employed day population lives in another municipality.	1998	6	6	7	8	8
	2003	5	6	7	8	8
	2008	1	5	6	8	8
	2013	-	5	7	7	7
8: Rural municipalities - municipalities with a population of less than 15 000 inhabitants in the largest urban area, very low commuting rate (less than 30%)	1998	5	5	6	6	7
	2003	4	4	5	6	6
	2008	2	4	5	6	6
	2013	-	4	5	5	6
9: Rural municipalities with a visitor industry – municipalities in rural area that fulfil at least two criteria for visitor industry, i.e. number of overnight stays, retail-, restaurant- or hotel turnover per head of population.	1998	2	1	1	2	2
	2003	1	1	2	2	2
	2008	1	1	1	2	2
	2013	-	1	1	1	2

^*^Percentages might not sum up to 100 due to rounding

^¤^ Total number of individuals in this age category including all years

^#^ The interaction test for age group 18–27 for differences in annual prevalence between 1998, 2003, and 2008 was statistically significant at p < 0.001

## Discussion

This study of a Swedish population-based sample of twins, illustrated regional differences in the annual prevalence of sustainable working life by age group, between sex, and for identical and fraternal same-sex twins. At the baseline, the annual prevalence of sustainable working life was high in large to medium size municipalities and changed a little or remained the same in the follow-up years. In regions with large to medium sized cities with good commuting with neighboring municipalities, the youngest age group (18–27 years) demonstrated high annual prevalence of sustainable working life from baseline in 1998 until 2008 and the difference between the subsequent years was statistically significant. No regional difference was found by sex or being identical or fraternal twin.

Previous studies of regional differences on SA/DP [8–10, 15–17, 40–42] indicate an interruption in an employment which is opposite to our definition of sustainable working life why the trends observed might lend some support to our findings. The underlying mechanisms for regional differences in sustainable working life might include composition of the population where younger individuals tend to move to urban municipalities for more attractive employment options; occupational composition, more labor-intensive work in rural municipalities; local attitudes towards SA and unemployment; differences in education levels; disparities in transport facilities; different social and socioeconomic factors at municipality level; and disparities in health care system, lack of health care and rehabilitation services in rural regions [14, 15, 17, 40–42].

Our finding of high annual prevalence of sustainable working life in the youngest age group is somewhat supported by earlier studies [17, 22, 41]. However, further studies are merited to investigate whether regions are associated with sustainable working life. We found no sex differences although women have shown to have an increased risk of SA/DP [17, 22, 41]. Furthermore, we found no differences between identical and same-sex fraternal twins which might be indicative for no familial effects, but this should be confirmed in future studies with co-twin control design.

The core idea of sustainable working life stands on creating favorable working and living conditions which keeps individuals in work throughout extended working life. Work characteristics and circumstances of an individual are influenced by a set of policies, regulations, and practices [26]. Understanding and identifying regional differences in sustainable working life might help in effective targeting of policies, regulations, and practices focused on promoting sustainable working life. Hence, our findings indicate regional differences in sustainable working life with low annual prevalence in rural municipalities with poor commuting and less employment option in their own municipality.

The main strength of this study was the large sample size (N = 81,231), several time points constituting together 15 years of follow-up, no drop out, and the availability of the high-quality data from Swedish national registries. Until now, studies of annual prevalence of sustainable working life have been rare although regional differences in SA/DP have been reported in the Nordic countries [19, 43–45]. A limitation of this study is the generalization of the results from Sweden to other countries. Sustainable working life was measured as not having interruptions of working life, which should be tested for inclusiveness in further studies and potentially complimented with assessment of other absences since we included only register data of SA/DP and unemployment. However, these findings might be applicable to other countries with similar welfare and social security i.e., Nordic countries, but less to other countries. Yet, although several time points can be considered as a strength, they may also comprise a limitation. Our data from 1998 to 2013 include changes in social insurance regulations [27, 28], and societal changes [31] e.g., the financial crisis in 2008 [29] or national policies [30]. Among limitations, studies with access both on twin and singleton data could test if the assumption of representativeness of regions and sustainable working life holds, although twins are known to be representative of the general population [46] and results based on SA/DP are consistent with singleton population [22, 40, 47]. Another limitation is that we utilized data from four time points, 1998 to 2013, why more frequent and especially more recent timepoints could be used since regional differences or their sociodemographic characteristics might not be stable [43, 48]. Further studies with more sophisticated approaches than descriptive statistics should tackle these issues.

### Electronic supplementary material

Below is the link to the electronic supplementary material.

Supplementary Material 1

## Data Availability

The data that support the findings of this study are available from the original sources: the Swedish Twin Registry, Statistics Sweden, Swedish Social Insurance Agency and the Swedish National Board of Health and Welfare. Restrictions apply to the availability of the data used in this study based on the Swedish Twin project Of Disability pension and Sickness absence (STODS), which were used with ethical permission for the current study and therefore are not publicly available.
