# Supplementary material for: Regional differences in annual prevalence of sustainable working life in Swedish twin cohort
Source: BMC Res Notes. 2023 Sep 21;16:228. doi: 10.1186/s13104-023-06503-y (PMC10515065; doi:10.1186/s13104-023-06503-y)
Supplement: Supplementary file 1 — Supplementary Material 1 [file 13104_2023_6503_MOESM1_ESM.docx]

Supplementary material

**Supplemental Table 1.** Follow up of individuals followed from the year 1998**:** Number of men and women with sustainable work life across different regions in Sweden. Information of individuals living in these regions and sustainable working life are from the years 1998, 2003, 2008 and 2013.

| **Classification of Swedish municipalities 2017 by Swedish Municipalities and Regions** | **Sex** | | | | | | | |
| --- | --- | --- | --- | --- | --- | --- | --- | --- |
|  | **Men** | | | | **Women** | | | |
|  | **1998**  (n = 29,035) | **2003**  (n = 27,863) | **2008**  (n = 26,056) | **2013**  (n = 22,018) | **1998**  (n = 27,220) | **2003**  (n = 24,988) | **2008**  (n = 23,343) | **2013**  (n = 19,998) |
| 1: Large cities - municipalities with a population of at least 200 000 inhabitants with at least 200 000 inhabitants in the largest urban area. | 15 | 16 | 16 | 16 | 16 | 17 | 17 | 17 |
| 2: Commuting municipalities near large cities – municipalities where more than 40 % of the working population commute to work in a large city or municipality near a large city. | 17 | 18 | 17 | 18 | 18 | 18 | 18 | 19 |
| 3: Medium-sized towns – municipalities with a population of at least 50 000 inhabitants with at least 40 000 inhabitants in the largest urban area. | 23 | 24 | 23 | 23 | 24 | 24 | 24 | 23 |
| 4: Commuting municipalities near medium-sized towns - municipalities where more than 40 % of the working population commute to work in a medium-sized town. | 9 | 9 | 9 | 9 | 8 | 8 | 8 | 9 |
| 5: Commuting municipalities with a low commuting rate near medium-sized towns - municipalities where less than 40 % of the working population commute to work in a medium-sized town. | 7 | 7 | 7 | 7 | 7 | 7 | 7 | 7 |
| 6: Small towns - municipalities with a population of at least 15 000 inhabitants in the largest urban area. | 15 | 14 | 14 | 14 | 14 | 13 | 14 | 14 |
| 7: Commuting municipalities near small towns - municipalities where more than 30 % of the working population commute to work in a small town/ urban area or more than 30 % of the employed day population lives in another municipality. | 7 | 7 | 7 | 6 | 6 | 6 | 6 | 6 |
| 8: Rural municipalities - municipalities with a population of less than 15 000 inhabitants in the largest urban area, very low commuting rate (less than 30 %) | 5 | 5 | 5 | 5 | 6 | 5 | 5 | 5 |
| 9: Rural municipalities with a visitor industry – municipalities in rural area that fulfil at least two criteria for visitor industry, i.e., number of overnight stays, retail-, restaurant- or hotel turnover per head of population. | 1 | 2 | 2 | 2 | 2 | 2 | 2 | 2 |


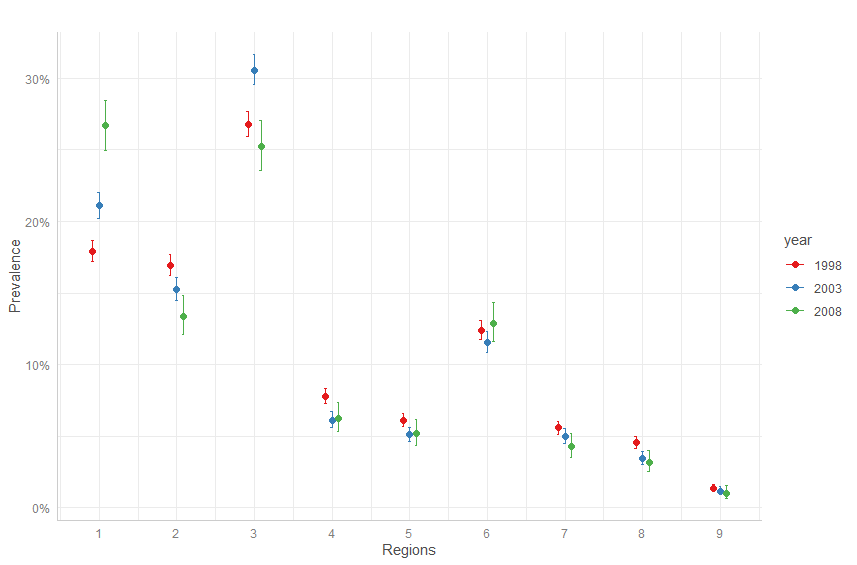


**Supplemental Figure 1.** Difference in sustainable working life between years in 18-27 years old age group within each region. Region 1 Large cities - municipalities with a population of at least 200 000 inhabitants with at least 200 000 inhabitants in the largest urban area, Region 2 Commuting municipalities near large cities – municipalities where more than 40 % of the working population commute to work in a large city or municipality near a large city, Region 3 Medium-sized towns – municipalities with a population of at least 50 000 inhabitants with at least 40 000 inhabitants in the largest urban area, Region 4 Commuting municipalities near medium-sized towns - municipalities where more than 40 % of the working population commute to work in a medium-sized town, Region 5 Commuting municipalities with a low commuting rate near medium-sized towns - municipalities where less than 40 % of the working population commute to work in a medium-sized town, Region 6 Small towns - municipalities with a population of at least 15 000 inhabitants in the largest urban area, Region 7 Commuting municipalities near small towns - municipalities where more than 30 % of the working population commute to work in a small town/ urban area or more than 30 % of the employed day population lives in another municipality, Region 8 Rural municipalities - municipalities with a population of less than 15 000 inhabitants in the largest urban area, very low commuting rate (less than 30 %), and Region 9 Rural municipalities with a visitor industry – municipalities in rural area that fulfil at least two criteria for visitor industry, i.e. number of overnight stays, retail-, restaurant- or hotel turnover per head of population
